# Boundaries and conditions of interpretation in multilingual and multicultural elderly healthcare

**DOI:** 10.1186/s12913-015-1124-5

**Published:** 2015-10-06

**Authors:** Emina Hadziabdic, Christina Lundin, Katarina Hjelm

**Affiliations:** Department of Social and Welfare Studies, Linköping University, SE-581 83 Linköping, Sweden; Department of Health and Caring Sciences, Faculty of Health and Life Sciences, Linnaeus University, SE- 351 95 Växjö, Sweden

**Keywords:** Ethnicity, Multilingual elderly healthcare, Interpretation, Interpretation practices, Organization

## Abstract

**Background:**

Elderly migrants who do not speak the official language of their host country have increased due to extensive international migration, and will further increase in the future. This entails major challenges to ensure good communication and avoid communication barriers that can be overcome by the use of adequate interpreter services. To our knowledge, there are no previous investigations on interpreting practices in multilingual elderly healthcare from different healthcare professionals’ perspectives. This study examines issues concerning communication and healthcare through a particular focus on interpretation between health professionals and patients of different ethnic and linguistic backgrounds. The central aim of the project is to explore interpretation practices in multilingual elderly healthcare.

**Methods:**

A purposive sample of 33 healthcare professionals with experience of using interpreters in community multilingual elderly healthcare. Data were collected between October 2013 and March 2014 by 18 individual and four focus group interviews and analysed with qualitative content analysis.

**Results:**

The main results showed that interpreting practice in multilingual elderly healthcare was closely linked to institutional, interpersonal and individual levels. On the organizational level, however, guidelines for arranging the use of interpreters at workplaces were lacking. Professional interpreters were used on predictable occasions planned long in advance, and bilingual healthcare staff and family members acting as interpreters were used at short notice in everyday caring situations on unpredictable occasions. The professional interpreter was perceived as a person who should interpret spoken language word-for-word and who should translate written information. Furthermore, the use of a professional interpreter was not adapted to the context of multilingual elderly healthcare.

**Conclusion:**

This study found that interpreter practice in multilingual elderly healthcare is embedded in the organizational environment and closely related to the individual’s language skills, cultural beliefs and socio-economic factors. In order to formulate interpreter practice in the context of multilingual elderly healthcare it is important to consider organizational framework and cultural competence, cultural health knowledge, beliefs and customs.

## Background

As result of global migration, there are growing multicultural and multilingual societies, and this trend is predicted to continue [1]. One in thirty individuals in the world is a migrant [1], and 15 % of the Swedish population are migrants, and the number of these aged over 65 is estimated to be around 217 000 [2]. Data on the number of elderly immigrants in Europe are limited and unsatisfactory, but the number of elderly migrants in Europe is estimated to increase [3]. Growing elderly migrant populations require healthcare staff to provide culturally and linguistically appropriate healthcare services [4]. Misunderstandings due to inappropriate communication between a healthcare provider and a patient are an increasing barrier to accessing healthcare [5], undermine trust in the quality of healthcare received [6] and are a risk to patient safety [7]. Further, it has been found that elderly migrants often receive healthcare in a lesser extent than native persons [8] and community healthcare lacks systematic cultural care assessment because of language problems [9]. Inappropriate communication can be overcome by the use of adequate interpreter services [10, 11] in order to deliver high-quality healthcare to all individuals irrespective of age, sex and ethnic origin [12, 13]. Interpreting practices are thus central in all healthcare, and therefore the focus of this study is to explore interpreting practices in multilingual elderly healthcare to develop interpreting practices which are affordable and accessible and also clinically relevant in relation to the particular needs that elderly people have due to ageing.

A previous study concerning interpreting practices in an elderly healthcare context is limited to investigating communication through professional interpreters on the spot [14]. The results described how the interpreter should interpret objectively and neutrally and also explain medical terms and other concepts when needed [14]. Previous studies have been found focusing on physicians [15, 16]; nurses in primary healthcare [17] and nurse radiographers in acute healthcare [18] and their experiences of communication with people who do not speak the same language; there is also a previous study focusing on different healthcare professionals’ experiences of the use of interpreters in primary and acute healthcare [19]. Physicians described how, in consultations through interpreters, they face obstacles in establishing optimal communication [15] and found all communication tasks more difficult when an interpreter was used [16]. Nurses reported experiences of the use of interpreters being related to the interpreter, the nurse, and the patient [17] and the need for an interpreter was associated with the type of examination [18]. The previous study [19] from different healthcare professionals’ perspective found that the interpreter as a person and the organizational framework were two main aspects affecting the use of interpreters. Another study [20] focused on identifying and exploring ways to improve communication across language barriers and on organizational routines for interpreter services in primary healthcare. According to recommendations in guidelines for interpreters in Sweden [21] the interpreter’s role is to do word-by-word interpretation. Further, satisfactory interpretation is achieved when the message and all its nuances are reproduced as correctly as possible. However, word-by-word interpretation has also been questioned previously [22, 23] as the interpreter affects communication by being present [24].

Insufficiency noted in the research is a lack of investigations concerning interpreting practices in multilingual elderly healthcare. Multilingual elderly healthcare differs in characteristics from primary and acute healthcare as it mainly concerns less structured activities in longstanding contacts with ageing people, possibly with impaired hearing, in contrast to highly structured consultations in primary healthcare delivered in a limited time frame.

### Theoretical framework

As previous research (11–17) has shown, interpretation is highly complex and thus not a straightforward question of translation. In this study we are particularly interested in the institutional context and how this influences interpretation practices.

The study’s main theoretical habitat is located in transcultural care and communication in an institutional setting. Transcultural care emphasizes the need to provide care based on an individual’s or group’s cultural beliefs, practices and values in order to avoid ethnocentric assessments [4]. It proceeds from an understanding that sees the main aim of caring as promoting or regaining health, and underlines that communication is central for caring. The term ethnicity is described as shared national and cultural traditions, and is used to describe group identification with a specific country or custom [4] and a “power relationship”, the relation between majority and migrant minorities, that constructs certain individuals/groups as non-associated and other-related [25]. The transcultural approach focuses not only on the patients’ cultural views but also stresses that we need to include the cultural understandings built into our medical institutions as well as the cultural background of the individual healthcare staff in order to provide culturally congruent healthcare [4, 26].

Communication in an institutional context differs from ordinary talk in which participants engage on fairly equal terms, and is characterized as asymmetrical when clients and professionals do not have the same aims, knowledge or resources. Professionals generally have an agenda with specific questions to be answered to gain information for decision-making [27]. Verbal and non-verbal communication is the important core of all healthcare encounters in transmitting messages clearly [4].

Organizational cultural competence involves the structures, policies, processes and strategies that work with healthcare organizations to ensure the delivery of high-quality healthcare and equitable outcomes to all individuals and that effectively challenge inequalities in healthcare experiences and results for different foreign-born groups [4]. Organizational routines are progressive structures that are often used as a way of fulfilling organizational work. The routines involves five actors: individual (identity, values and goals, competence); interpersonal (social skills, personality, power and influence); organizational (technological structures, coordination structures and cultural structures); institutional (regulative posts, normative posts, cultural-cognitive posts); and environmental (economic/political climate, legislative constraints, demographic changes, technological developments). These actors cooperate dynamically and equally with one another [28, 29].

## Aim

This study examines issues concerning communication and healthcare through a particular focus on interpretation between health professionals and patients of different ethnic and linguistic backgrounds. The central aim of the project is to explore interpretation practices in multilingual elderly healthcare. Leading research questions for the study include:

1. How and when does interpretation become an issue in multilingual elderly healthcare?
2. How is interpretation conceptualized, e.g., what is supposed to be interpreted?
3. What are the implications of different interpretation practices (such as by telephone, in person, the use of family or bilingual healthcare staff)?
4. What are the challenges concerning interpretation for improving equal healthcare and patient involvement?

## Method

### Design

This study is an explorative and descriptive study using semi-structured individual interviews and focus group interviews to understand the reality of interpretation practices in multilingual elderly healthcare from different healthcare professionals’ perspective [30]. The individual and focus group interviews were used to generate data of the depth and by the interaction in the group to reach a person’s more or less unconscious [31, 32]. Further, focus group interviews have the strength of opening up for discussions where different perspectives are ventilated [32].

### Setting

Two municipalities located in two different migrant-dense regions in Sweden were studied. Municipality 1 served approximately 15 657 elderly persons (above 65 years), of whom 1361 were foreign-born. Municipality 2 served approximately 24 607 elderly persons (above 65 years), of whom 5904 were foreign-born [2].

The studied areas follow the Swedish legislation: the Management Act [33], the Social Services Act (1982) [34] and the Health and Medical Services Act [13]. According to the Management Act [33], an interpreter should be used in all contacts with public authorities for individuals who have communication barriers. The Social Services Act (1982) [34] declares that elderly have the right to receive public service and help at all stages of life, and the Health and Medical Services Act [13] states that healthcare and medical services aim to preserve good quality of health among the entire population and to provide care on equal terms for everyone, irrespective of race, religion or ethnic origin.

The aims of care of the elderly in Sweden are to guarantee financial security, good housing, and service and care according to individual needs. Municipalities are legally obliged to meet the social service and housing needs of the elderly in private homes, service apartments and nursing homes [35]. Social welfare includes services such as food distribution, administration of security alarms, organization of social activities, assistance with eating and personal hygiene, and general house cleaning. Service apartments function as private homes and services and care required are offered by home care personnel. Nursing homes have 24-h nursing surveillance. During the last few years, the care of older people has shifted from long-term care institutions to community-based care facilities and private homes. This has meant that the older people receiving care in either homes or nursing homes have more disabilities and complex health needs than was previously [35].

In the studied municipalities the interpreter service agencies are run by private enterprises outside healthcare. Institutions in the public sector follow the Public Procurement Act [36] concerning specification of how the contract should be designed and what requirements are of major importance to guarantee the quality of the interpretation service. Procurement is done by the provider of interpreting services, and the Public Procurement Act [36] specifies that contracts must be signed with the most economically advantageous interpreter agency after an assessment based on criteria of price and quality. In multilingual elderly healthcare, the responsibility for arranging an interpreter lies with the municipalities. The professional interpreter’s attitude includes word-for-word translating, being neutral, ensuring confidentiality and not making any other statements unrelated to the situation [21].

### Procedure and participants

A purposive sample was chosen to ensure maximum variation [30] in age, gender and different healthcare professions with frequent communication in their daily work in multilingual elderly healthcare with elderly persons who do not speak Swedish.

Managers in different elderly healthcare institutions located in a migrant-dense region were contacted by telephone by the authors, EH and CL, to inform about the study and to obtain permission for implementation. After approval, the managers were requested to invite persons to participate who had experience of communication with non-Swedish speaking elderly persons and represented different healthcare professions. A time was set for information meetings where the participants received verbal and written information about the study from EH and CL. Those interested in participating gave their contact details and were contacted to set a time and place for the interview. The snowball method [30] was also used to try to who had not attended these meetings. The sampling started when the first author asked early informants to refer to other people who met the eligibility criteria and then continued on the basis of informants’ referrals. Eight participants were recruited in this way.

The study included 33 persons (31 females and 2 males, see Table 1) who had experience of work in multilingual elderly healthcare from 3 to 40 years (Md 22 years).

**Table 1:** Characteristics of the study population

Variable	Persons (*N* = 33)
Gender *(n)*	
Female	31
Male	2
Age (years)^a^	45 (25–63 years)
25–35 years	5
36–45 years	12
46–55 years	12
56–65 years	4
Work experience in current position (years)^a^	4 (6 months–13 years)
0.5–5 years	15
6–10 years	7
10–13 years	7
Missing information	4
Work experience in multilingual elderly healthcare (years)^a^	22 (3–40 years)
3–13 years	9
14–24 years	8
25–35 years	8
36–40 years	4
Missing information	4
Professional level	
Registered nurse	12
Physiotherapist	1
Occupational therapist	2
Assistance officer	2
Recreation leader	1
Assistant nurse	13
Missing information	2
Sector	
Community home elderly care	18
Nursing home	9
Nursing home with dementia patients	2
Municipality	3
Position in hierarchy	
Employee	26
Medically responsible nurse	2
Middle manager	1
Manager	4

^a^Values are median (range)

### Data collection

Data were collected between October 2013 and March 2104. By interviews, individually and in focus groups, with an interview guide developed based on previous literature [19, 37–39] and focused on experiences of routines, guidelines and existing preconceptions among healthcare staff about what, why and how interpretation should take place.

In accordance with participants’ wishes, all 18 individual and four focus group interviews were conducted at their workplaces. All interviews were held in secluded venues chosen by the informants. The moderator (EH, sometimes CL) guided the focus group and the assistant moderator (CL, sometimes EH) was present to take detailed observational notes. The size of focus groups was three to four participants and informants were a fairly homogeneous group in terms of profession and gender, in order to promote a comfortable group dynamic [32]. As a result, the focus group interaction was lively and the discussions lasted about 60–90 min. Individual interviews were conducted by authors EH and CL. Communication in individual interviews proceeded in an uncomplicated, free-flowing way and lasted about 30–60 min. All interviews were audiotaped and transcribed by a professional secretary and then analysed.

### Data analyses

In order to identify patterns in the data and to discover relationships between experiences, inductive qualitative content analysis was used to analyse individual and focus group interviews [40].

Directly after the focus group interviews, the interviewers noted the content of what participants had discussed and the interaction in the group [32]. Then, all individual and focus group data were read thoroughly several times to achieve a sense of the whole. The texts were then broken into smaller textual units. The next step was to search for similarities and patterns to develop sub-categories and categories from the context of the textual units. During the whole analysis process, authors searched for regularities, contradictions, similarities and patterns, returning to the data analysis and rereading all the transcripts until no new information was found. Categories were developed, modified and refined until an acceptable system was recognized. In naming categories, concepts as close as possible to the text were used. Analysis of data proceeded until no new information was obtained [40].

To enhance the trustworthiness of this study, the following steps described by Patton [30] were taken. Credibility was confirmed by the first and second author conducting and analysing the data. The codes and categories were also reviewed and assessed for relevance by the co-authors having research and practical experience in healthcare. Confirmability was ensured by having categories accompanied by literal quotations and by naming categories as closely as possible to the text. Dependability was confirmed by describing the investigation process as clearly as possible [30].

### Ethical considerations

Swedish law [41] and ethical considerations according to the Declaration of Helsinki [12] were followed. Written informed consent was obtained from the participants before the interviews started. The confidentiality of the participants’ data was ensured by having the tapes and transcripts anonymized and coded by number. The analysis and presentation of the data were done in a way that concealed the participants’ identity. Participation was voluntary and participants could withdraw from the study at any time without explanation. All data were stored in a locked space to which only the first author (EH) had access [12]. Approval by an official research ethics committee was not required as the investigation aim posed no physical or mental risk to the informants and did not treat informants’ personal data. Thus, the study has been implemented in accordance with Swedish law [41] and the Helsinki Declaration [12].

## Results

Five categories emerged from the analysis of the interviews (see Table 2).

**Table 2:** Overview of categories with subcategories analysed from interviews by healthcare staff working in multilingual elderly healthcare

Category	Subcategories
Organization of interpreter practice in multilingual elderly healthcare in accordance with the Public Procurement Act	1) Existing guidelines at the workplace concerning the use of interpreters
	2) The lack of guidelines in multilingual elderly healthcare on the use of interpreters in the organization of daily healthcare led to limited use of professional interpreters
Organization of interpreter practice in daily work	1) Use of professional interpreters for interpretation in daily work *multilingual elderly healthcare* occurs in care planning and problematic circumstances, and requires planning long in advance during office hours
	2) Use of informal interpreters in *multilingual elderly healthcare* everyday caring situations and in unpredictable requirements at short notice even outside office hours
The professional interpreter’s role in communication in everyday multilingual elderly healthcare	1) The interpreter’ role as spokesperson and translating written information
	2) The interpreter’s role including competence in elderly illnesses
Healthcare and communication practices for non-Swedish speaking elderly	1) Challenges in providing healthcare for elderly require planning far in advance
	2) Challenges in communication through an interpreter, such as the communication process taking longer and feelings of insecurity as to whether or not to translate word-for-word
Wishes for future development of interpreter practice in multilingual elderly healthcare	1) Making the interpreter policy/routines easily available and understandable to all staff in multilingual elderly healthcare
	2) Simplifying and developing the procedure for the use of interpreters at the workplace
	3) Training of healthcare staff in using and communicating through interpreters
	4) Developing the training for professional interpreters for interpretation in multilingual elderly healthcare

## Organization of interpreter practice in multilingual elderly healthcare in accordance with the Public Procurement Act

### Existing guidelines at the workplace concerning use of interpreters

There were considerable similarities between the workplaces in how they had developed policies for providing professional and informal (family members or bilingual) interpreters for multilingual elderly healthcare. The workplaces had established policies that included access to an interpreter agency which municipalities had ensured through the Public Procurement Act. The guidelines included information on the respective municipality’s website, the name and contact details of the interpreter agency that the municipalities have a contract with. Informants described how information was missing in the guidelines about the procedures and implementation of the use of interpreters, such as where, how and how much and what type of interpreter to be used in different situations.

*…the contracts say more about what is procured, which company, the time frame, the service we expect … to some extent invoicing or such matters, but not the level of detail – this is what you should do if you are going to use an interpreter. … not guidelines, that we have a document describing how it should be from decision to implementation … more perhaps stating that if it happens in the implementation of the plan that people don’t speak or understand then you should ensure the use of an interpreter (I:11).*

Managers and assistance officers described their organization of the use of interpreters more in terms of efficiency, accessibility, professionalism and equity for the elderly. In contrast, other staff put more emphasis on a personal atmosphere and continuity in the use of interpreters and the need to be friendly, caring and appreciated. The personality and commitment of the staff (assistant nurse, nurse, physiotherapist and occupational therapist) was often highlighted by staff, and organizational values were personalized in terms of the views or attitudes prevailing in the workplace.

### The lack of guidelines in multilingual elderly healthcare on the use of interpreters in the organization of daily healthcare led to limited use of professional interpreters

Most informants found the limited use of a professional interpreter service inefficient because of lack of guidelines in the organization on how to use an interpreter in the daily elderly healthcare. Arranging the use of professional interpreters required long planning, limited access to the professional interpreters who could interpret at short notice and good access to bilingual healthcare staff who could be used as interpreters at short notice.

*it takes quite a lot of planning then (with professional interpreters), there are often emergency situations … and … give this treatment immediately, that something acute has happened … it’s good that we also have some staff who speak Bosnian because we have many from Bosnia here and so you end up using them (I:6).*

Guidelines concerning responsibility for arranging the interpreter service were lacking, and the division of responsibility for this between the healthcare professions varied from one workplace to another. Mostly it was done by assistance officers using the telephone booking form; sometimes the nurses, physiotherapists, occupational therapists and the head of unit could book a professional interpreter by phone. The assistant nurses were not aware of the existing guidelines at the workplaces concerning the use of interpreters or how to book available interpreter service. They enlisted family members or bilingual healthcare staff as interpreters when they needed to talk through an interpreter. The informants described how in most cases it was the healthcare staff needs that determined the use of interpreters. It was also found that in some cases it was the patients’ wishes that determined the use of interpreters at the workplace.

*those who are involved, who help to make the decision but … if it’s us (care staff), the one who calls the meeting is the one who makes the final decision and if it’s care planning it’s the assistance officer who … who gets to decide (I:13).*

## Organization of interpreter practice in daily work

### Use of professional interpreters for interpretation in daily work in multilingual elderly healthcare occurs in care planning and problematic circumstances, and requires planning long in advance during office hours

Informants felt that the availability of an adequate interpreter agency was good. They also stated that the use of interpreters depended on needs that arose in relation to the healthcare situation, on the personal feeling that healthcare staff got in the healthcare encounter and on the easy availability of different types of interpreters. Professional interpreters were used at scheduled meetings such as doctor’s appointments and the meeting of the local authority to decide on care planning. In contrast, family members and bilingual healthcare staff were used during the everyday and emergency healthcare situations.

Informants described the inflexibility of the interpreting service, which restricted its service to office hours and required long planning in advance by healthcare staff. Healthcare staff mentioned several problems in the effective use of professional interpreters in day-to-day work. Thus, the arranging of professional interpreters was not adapted to easy availability at short notice but required longer planning, which was perceived as a problem.

*… if we order one but it takes some time before the interpreter can get here. We can’t have that in our everyday work (I:2).*

Another frequently expressed problem regarding the use of professional interpreters was elderly persons’ own expectations, behaviour and illness. Some elderly persons may feel uncomfortable with strangers in the healthcare encounter, and also with a professional interpreter who does not share the same country of origin and dialect. It could also be that some focused more on making contact with the professional interpreter than with healthcare staff, who felt left out. Informants described how elderly people suffering from dementia and/or aphasia need to have someone they recognize, as it can be more difficult with unknown people such as professional interpreters.

*… some disadvantages because of … Then a completely unknown person comes, and there can be people who feel a bit uncomfortable with that … for example, the assistance officer had phoned for an interpreter and an interpreter came, from Serbia or somewhere … he was almost regarded as an enemy (I:11).*

Participants perceived several difficulties when using telephone interpretation in multilingual elderly healthcare: it requires a certain functional ability in the older persons, who are often unable to follow the body language and feel discomfort about using technical equipment. Further, it was felt that the technical problems were also related to the lack of good equipment at the workplace ensuring good sound quality on the telephone.

*Some people can’t even talk on a telephone. They don’t know what it is, they don’t understand, they can’t hear. Their hearing is far too bad to manage it, it’s a huge problem (I:10).*

### Use of informal interpreters in multilingual elderly healthcare everyday caring situations and in unpredictable requirements at short notice even outside office hours

A common feature of workplaces in the municipalities was the number of languages spoken by healthcare staff. Workplaces habitually employed bilingual healthcare staffs who were fluent in the main languages represented in their district. Bilingual staffs were used for interpreting in everyday caring situations and for unpredictable requirements at short notice. At the workplaces, the norm for bilingual healthcare staff was to offer their language skills as needed for interpreting, with the advantage of being unpaid, which saved money for the municipalities. Other benefits were their familiarity with the duties, roles and routines that exist at the workplace, but also with elderly persons, leading to feelings of security and confidence. Further, using bilingual healthcare staff for interpreting could also be perceived as negative because the elderly person might feel dependent on the staff, with the consequence that they might be unwilling to disclose certain things, especially concerning complaints about the care received.

A few bilingual informants noticed that in some cases when they acted as interpreters, the elderly persons expected that healthcare staff should perform other duties not included in their work role, and the elderly persons’ expectations that specific bilingual healthcare staff will always be available for their needs regardless of practical difficulties in meeting those requirements. Further, interpreting was not part of their work assessment and they felt exploited as interpreters by employers, and some healthcare situations were difficult to interpret because they could be blamed for holding the ideas expressed by other professions.

*…, who you’ve sometimes phoned for outside working hours and who was more than willing to come when someone was upset (I:1).*

*… bilingual staff … saved a lot of money for the municipality since we didn’t order an interpreter …. there must be bilingual staff, right, that’s a part of helping them (I:3).*

Mostly informants felt that family members as interpreters were the second best choice, as they fitted in with existing routines, ways of working in multilingual elderly healthcare and the family members’ role as an integral part of the caring relationship and their involvement in the care of elderly. In some cases, using the family members as interpreters had been associated with poorer-quality interpretation because of the negative emotional influence on family members, especially when interpreting negative and/or sensitive news.

*And then also, when it’s a family member, they don’t always know the language of officialdom so there can be mistakes or maybe the words are just twisted a bit. I can imagine that some other matters such as other cultures – that some things are taboo, you don’t speak certain words (I:12).*

## The professional interpreter’s role in communication in everyday multilingual elderly healthcare

### The interpreter’s role as a mouthpiece and translating written information

The most commonly accepted professional interpreter role was as a “mouthpiece” who conveys spoken language verbatim, ensuring understanding and being confidential and impartial, but also translating the written information used in daily elderly care, such as menus. Some informants discussed the professional interpreter’s role of being accurate but also ensuring understanding in multilingual elderly healthcare. They stressed that it was important that the professional interpreter adapts the use of language to suit elderly people’s preferences. Some elderly persons had illnesses such as dementia and/or aphasia, low levels of education and literacy and unfamiliarity with the Swedish healthcare system, which led to a struggle to understand what was said when it was interpreted word-for-word.

*The interpreter should serve language between the old person and, for instance, the assistance officer, and the interpreter’s duty is not actually to interfere in the conversation, he should be an object, be alongside and preferably not be visible, he should just be there with his words, no gestures, no opinions but should be as objective as possible so that the dialogue is between the user and the municipality (I:15).*

### The interpreter’s role including competence in elderly illnesses

Informants said that it was important that the professional interpreters saw their role as including competence in the most common elderly illnesses and asking healthcare professionals to clarify the terminology, paraphrase or use simple Swedish language if they did not understand.

*… then the interpreter must also have a good understanding of dementia)…*

*You (the interpreter) can’t start laughing. Or you can’t just ask a question and expect, you can’t translate directly, it can end up seriously wrong (I:10).*

The role of a practical and informative guide in the healthcare system was not viewed as a part of the professional interpreters’ duty according to the informants.

*They (interpreters) should interpret and there shouldn’t be too much talk in between (I:14).*

## Healthcare and communication practices for non-Swedish speaking elderly

### Challenges in providing healthcare for elderly require planning far in advance

Three different experiences concerning non-Swedish multilingual elderly healthcare were expressed: some informants felt that healthcare is equal irrespective of language; others thought that healthcare is unequal depending on language; and some informants said that healthcare is better for non-Swedish speaking elderly. Informants emphasized that the community-based multilingual elderly healthcare includes much more than interpretation on special occasions. Multilingual elderly healthcare is not emergency healthcare; it requires long planning and different kinds of engagement of healthcare staff. It is about conversation, information, activities and social network in daily life, which the elderly are entitled to receive regardless of the language they speak in order to be able to be involved in the healthcare. Healthcare staff found the care of non-Swedish elderly very time-consuming, with an increased workload, because they used body language to communicate and to arrange different interpreters to ensure that elderly have understood correctly. Further, the healthcare staff felt emotionally affected and frustrated by the stress developed when being unable to communicate with their patients.

*we (elderly care) don’t give emergency care. We are stable and so we have a good chance to get to know the person, that’s more important than all the tablets maybe. We build up relationships and then you have to be able to communicate with language, so it’s obvious that my view of caring is being able to talk (I:19).*

Some informants said that many non-Swedish-speaking elderly persons are not as involved in the care as Swedish-speaking people because of their lack of language skills, with the result that not all information is available or available information is misunderstood. Further, they do not have the same opportunities to be active in daily social activities and feel neglected by healthcare staff because of their lack of language skills.

### Challenges in communication through an interpreter, such as the communication process taking longer and feelings of insecurity as to whether or not to translate word-for-word

Informants noted a number of challenges in communication and interpretation in multilingual elderly healthcare, as it is an interaction between three parties and the communication process takes longer in the healthcare encounter. Communication through an interpreter was considered more difficult because it compounded the problem of sending messages clearly and the lack of nuances of language and communication. These included feelings of insecurity as to whether or not to translate word-for-word what is said because it is impossible to guarantee that the information will be correctly interpreted.

*It’s harder to talk via an interpreter … demands something from us (care staff) too, because it takes much longer and you can’t say much … at a time … then you have to find a simpler way to explain (I:4:).*

## Wishes for future development of interpreter practice in multilingual elderly healthcare

### Making the interpreter policy/routines easily available and understandable to all staff in multilingual elderly healthcare

One of the most commonly suggested improvements was to make the policy on the use of interpreters, including guidelines concerning the use of different types of interpreters and modes of interpreting, accessible and easy to understand to all employers at the workplace. They suggested faster and easier access to the interpreter service, especially for unpredictable requirements at short notice, and an open interpreter service also after office hours. This could be done on an organizational level, making the policy accessible and readable on the workplace’s internal website and having a person with responsibility to continuously educate healthcare staff about the rules and procedures concerning interpreter use.

*… that you adopt this policy to the activity in such cases so that it’s easily accessible and easy to understand (I:9).*

It was emphasized that one could facilitate healthcare and interpreter use by making the information regarding the aims of multilingual elderly care in Sweden available to non-Swedish-speaking persons and their relatives so that they can understand the structure of elderly care and what elderly can expect in the field of multilingual elderly healthcare, to avoid imposing extra duties on bilingual healthcare staff which are not a part of their work assignment.

### Simplifying and developing the procedure for the use of interpreters at the workplace

Participants expressed a need to simplify the work procedure for using professional interpreters. Participants provided suggestions to optimize the use of interpreters: (i) ensuring good access to interpreters with particular language skills, especially to be taken into consideration when planning for groups of elderly migrants who are expected to increase in future geriatric healthcare; (ii) trying a different approach and not only interpreting during short sequences planned far in advance; (iii) developing the technical interpreting tools that can be used by healthcare staff in the daily caring encounter; and (iv) employing more healthcare staff with different cultural backgrounds to increase the possibility of easy access to interpreters at short notice.

*… an area that needs to be developed. More interpreters, … a worry for the future … and then we have the peak of people needing elderly care … to get a language machine to interpret short sequences at health centres or some clinic is different from doing it in everyday life (in elderly care). … that you must have a different approach than in the short visits … use technology in communication and the encounter (I:17).*

A few informants also wished to see interpreters who could help to translate written information in elderly daily healthcare, such as the menu, information about chronic illness, activities in multilingual elderly healthcare, but also better planning regarding the placement of non-Swedish elderly persons in the proper department, with bilingual healthcare staff available in order to communicate in daily life and not just on special occasions.

### Training of healthcare staff in using and communicating through interpreters

Informants reported a need for ongoing education in how to use and communicate through professional interpreters. They desired training on an individual level in basic practical access to professional services procured by the municipality and increased awareness of healthcare encounters requiring the use of an interpreter. There was also a desire for ongoing training in (i) how to communicate through interpreters in order to ensure adequate communication; and (ii) how the interpreters’ personal characteristics such as gender, ethnic and religious origin and dialect may affect the personal relationship between the elderly and the professional interpreter, which in turn may disturb the communication flow.

*… that you bring in the need for interpreters into your own way of thinking perhaps… (I:17).*

### Developing the training for professional interpreters for interpretation in multilingual elderly healthcare

From the healthcare staff’s point of view better training of interpreters was desired, both in professional attitude and in interpreting technique, particularly to interpret objectively and literally, to facilitate the use of interpreters. Further, some emphasized changes in interpreter training, such as reduced knowledge requirements to be easier to enter interpreter training programmes, and also better employment conditions for professional interpreters.

*… that they (professional interpreters) should interpret word-for-word what is actually said (I:5).*

## Discussion

In summary, the main result showed that, although there is an established law in multilingual elderly healthcare concerning the availability of an interpreter agency with which the municipalities have an agreement according to the Swedish Management Act [33] and the Public Procurement Act [36], guidelines were lacking in the organizational content concerning the use of interpreters, the responsibility for arranging an interpreter service and the procedure for booking an interpreter. The type of interpreter was a decision made by participants in everyday healthcare. The participants described two strategies for dealing with such situations: professional interpreters were used in situations concerning medical issues and care planning issues, and non-professional interpreters such as bilingual healthcare staff and family members were used in everyday caring issues and for unpredictable requirements at short notice. Thus, the use of professional interpreter was not adapted to the context of multilingual elderly healthcare because multilingual elderly healthcare is a complex field involving elderly people’s needs, quality of life and treatment planning far in advance and not only on special occasions as in primary and emergency healthcare. The professional interpreter’s role, it was felt, should be to interpret spoken language word-for-word and also to translate written information. Finally, some participants found difficulties in providing good-quality healthcare to migrants and communicating through an interpreter, as this entailed a greater workload for healthcare staff.

The first new finding, not previously described, was that the municipalities followed the existing law that included the contract about access to a certain interpreter agency but that there was a lack of guidelines concerning the procedure for using an interpreter. At the institutional level, the workplaces follow the Swedish legislation in the Management Act [33] and the Social Services Act (1982) [34]. However, in the daily multilingual elderly healthcare the arranging of the interpreter practice mostly took place on the participants’ individual and interpersonal level, influenced by their perception of their own linguistic and healthcare expertise and that of others, how they perceived their position and authority to make the decision about interpreting. The healthcare staff mostly relied on and preferred bilingual colleagues and family members as interpreters to fill the communication gaps between them, because they fitted better with existing routines and ways of working in the institution. This result contrasted with previous findings from different healthcare staff [19] and migrants with different cultural and language background [37, 42, 43] stating that they preferred using professional interpreters in healthcare encounters. Previously, many positive aspects have been described in using bilingual healthcare staff in everyday matters [37, 39, 44] and having family members as interpreters, such as feelings of trust [45–47]. In contrast, many negative situations have been described in using bilingual healthcare staff and family members, such as communication barriers because of non-neutrality and confidentiality problems [48, 49], limited knowledge of medical terminology and inadequate language skills [19, 50]. However, this study found something not described previously, that the bilingual healthcare staff described negative experiences of workplaces organized in such a way that they were used as interpreters, as they felt exploited by both employers (managers) and patients because interpreting was not regulated as a part of their work assignment. The development and delivery of effective routines depends on structuring devices, people and organizational learning [28]. Employee action is motivated by will and intention [29], and factors such as knowledge, confidence, experience, values, expectations, access to resources, and orientation are included when people select a routine [51]. As previously documented, arranging an interpreter can be problematic even when a policy is available, on both the institutional and the organizational level, concerning the procedures for and implementation of the use of an interpreter service [38]. Thus, our findings indicate the need for development at the organizational levels of multilingual elderly healthcare in order to provide interpreting, to give access a professional interpreter service and not only rely on bilingual healthcare staff and family members as interpreters. This is in order to achieve high-quality multilingual elderly healthcare, satisfaction and goal fulfilment in the multicultural workplace [4, 20, 29].

The second new finding, not previously described, was that elderly person’s expectations, behaviour and illness, according to the healthcare staff interviewed, affected the use and role of the interpreter. This aspect was valuable for the use of interpreters in the area of multilingual elderly healthcare with its composition of elderly persons’ specific needs, illnesses and treatment. Elderly people are living to an advanced age, with the result that many suffer from illness and become dependent on help from others [35]. Previously, the interpreter’s role was to act as a translation machine providing accurate and neutral communication [52], but the role of the interpreter has also been questioned [22, 23] and described as complex and extended to function as advocacy and a bridge between cultures [53–55]. Thus, the interpreter affects the circumstances of the conversation [24] with communication tending to be partial and centred on healthcare staff [56]. This study confirms that the interpretation situation in the field of multilingual elderly healthcare is a social and structural act, and as such is multifaceted and dependent [4] on social context, power relations, cultural beliefs, religious and traditional values, the physiology of illness and treatment, through the interpersonal dynamics of the healthcare encounter, to the social, institutional and governmental policies and practices [4, 26]. This highlights the need to improve the interpreter situation in multilingual elderly healthcare, considering not only the individual’s language skills, cultural beliefs, and socio-economics factors but also organizational cultural awareness [4].

The third new finding of this study was the challenges in providing healthcare for the elderly migrants and challenges in communication when healthcare staff used interpreters in multilingual elderly healthcare*.* Disparities between non-Swedish-speaking and Swedish-speaking elderly were often related to the fact that the former did not have the same opportunity to be equally involved in the healthcare because of lack of language. Both equal healthcare and patient involvement have been key issues in health policy development and are essential for patient safety as well as quality of healthcare [13, 57]. However, recent overviews of existing knowledge have shown that ethnicity is an underdeveloped perspective in both policy and practice [58], and has been reported as a barrier to the quality of healthcare among people of migrant background [59, 60]. The ethnicity perspective needs to be recognized in the context of multilingual elderly healthcare in order to avoid assumptions about ethnicity as a set of fixed cultural properties and instead to provide healthcare on the assumption that the patients are heterogeneous groups with diverse social aims and interests and that there are organized processes that prevent equal healthcare and patient involvement in healthcare [4, 25]. The results emphasize the need for better organization, making interpreter routines simpler, easily available and understandable, and for the development of specific training for healthcare professionals and professional interpreters working in the field of multilingual elderly healthcare. Carefully conducted interpretation requires, knowledge about the factors that influence interpreting practice and it takes good planning with carefully considered choices concerning the right kind of interpreter, the mode of interpretation and the patient’s preferences for the interpretation in order to promote communication, thus increasing quality of healthcare [48].

The strength of this study was that it included several different professions in order to investigate the experiences of those working in multilingual elderly healthcare, studied by different qualitative methods of data collection such as individual and focus group interviews [30]. Using the individual interviews avoided concerns that some informants were uncomfortable about expressing their experiences in front of a manager or group. The focus group interviews, on the other hand, stimulated participants to react to what was said by others, thereby leading to deeper expressions of experiences [32]. It has been stated that the same information can be obtained by individual interviews as by focus group interviews, although much more time is needed in the former [61]. Two authors conducting the majority of the individual interviews separately could be seen as threat to the quality of the interviews [30, 31]. To avoid this, data were collected using semi-structured interviews based on an interview guide to ensure that all interviews followed a standardized structure concerning topics aimed to answer the aim of the study [30, 31].

Another strength was using snowball sampling, giving the possibility to make the investigation cost-effective and practical, by spending less time screening informants to determine whether they were appropriate for the study and less time establishing a trusting relationship with new participants [30]. However, a weakness of this could be that participants in the study might be restricted to limited networks opportunities, and the quality of the recommendations may be affected by whether the recommending informants trusted the researcher and actually wanted to join the study [30]. Therefore, the recruitment procedure started by contacting managers in different multilingual elderly healthcare services located in migrant-dense regions to arrange the information meeting. At the information meetings, the first author asked for voluntary participants who could be recruited to the study in order to avoid weakness in data. This gave the opportunity to recruit an additional eight informants.

The aim of the study was to explore the content of interpreters’ practice from the perspective of different healthcare professionals in the clinical area of multilingual elderly healthcare. Findings from this investigation can contribute to improved knowledge of the organizational context in which the elderly clinical practice takes place [30, 40] and the results can be transferred to other contexts with similar characteristics, as several professional groups in two different municipalities had homogeneous experiences [30].

## Conclusion

This study found that experiences in multilingual elderly healthcare for migrants are embedded in the organizational environment of the interpreting practice, including the availability of laws, policy and guidelines, and closely related to the individual’s language skills, cultural beliefs and socio-economics factors. Furthermore, the use of professional interpreters was not adapted to the context of multilingual elderly healthcare; multilingual elderly healthcare was described as a complex field as regards the complexity of elderly people’s needs, quality of life and treatment planning far in advance and not only on special occasions such as in emergency healthcare. This study highlights the importance of improvements in the multilingual elderly healthcare considering the dynamics of organizational routines, organizational cultural awareness and individual cultural health knowledge, beliefs and customs when formulating interpretation practice to improve individual-centred healthcare for elderly migrants.
